# Retraining walking over ground in a powered exoskeleton after spinal cord injury: a prospective cohort study to examine functional gains and neuroplasticity

**DOI:** 10.1186/s12984-019-0585-x

**Published:** 2019-11-21

**Authors:** Atif S. Khan, Donna C. Livingstone, Caitlin L. Hurd, Jennifer Duchcherer, John E. Misiaszek, Monica A. Gorassini, Patricia J. Manns, Jaynie F. Yang

**Affiliations:** 1 Neuroscience and Mental Health Institute, University of Alberta, Edmonton, Alberta Canada; 2 Department of Physical Therapy, University of Alberta, 2-50 Corbett Hall, Edmonton, AB T6G 2G4 Canada; 3 Biomedical Engineering, University of Alberta, Edmonton, Alberta Canada; 4 Occupational Therapy, University of Alberta, Edmonton, Alberta Canada

**Keywords:** Powered exoskeleton, Spinal cord injury, Walking, Rehabilitation, Locomotion, Neuroplasticity

## Abstract

**Background:**

Powered exoskeletons provide a way to stand and walk for people with severe spinal cord injury. Here, we used the ReWalk exoskeleton to determine the training dosage required for walking proficiency, the sensory and motor changes in the nervous system with training, and the functionality of the device in a home-like environment.

**Methods:**

Participants with chronic (> 1 yr) motor complete or incomplete spinal cord injury, who were primarily wheelchair users, were trained to walk in the ReWalk for 12 weeks. Measures were taken before, during, immediately after, and 2–3 months after training. Measures included walking progression, sitting balance, skin sensation, spasticity, and strength of the corticospinal tracts.

**Results:**

Twelve participants were enrolled with 10 completing training. *Training progression and walking ability:* The progression in training indicated about 45 sessions to reach 80% of final performance in training. By the end of training, participants walked at speeds of 0.28–0.60 m/s, and distances of 0.74–1.97 km in 1 h. The effort of walking was about 3.3 times that for manual wheelchair propulsion. One non-walker with an incomplete injury became a walker without the ReWalk after training. *Sensory and motor measures*: Sitting balance was improved in some, as seen from the limits of stability and sway speed. Neuropathic pain showed no long term changes. Change in spasticity was mixed with suggestion of differences between those with high versus low spasticity prior to training. The strength of motor pathways from the brain to back extensor muscles remained unchanged. *Adverse events:* Minor adverse events were encountered by the participants and trainer (skin abrasions, non-injurious falls). *Field testing*: The majority of participants could walk on uneven surfaces outdoors. Some limitations were encountered in home-like environments.

**Conclusion:**

For individuals with severe SCI, walking proficiency in the ReWalk requires about 45 sessions of training. The training was accompanied by functional improvements in some, especially in people with incomplete injuries.

**Trial registration:**

NCT02322125 Registered 22 December 2014.

**Electronic supplementary material:**

The online version of this article (10.1186/s12984-019-0585-x) contains supplementary material, which is available to authorized users.

## Background

Restoring walking is of high priority for individuals with thoracic spinal cord injury (SCI) [1–3]. Treadmill and over ground training improved functional walking for those with sufficient residual strength in the lower extremities, but is much less effective for those with severe injuries [4–7]. Assistive devices such as reciprocating gait orthoses with or without functional electrical stimulation can restore standing and walking for severely injured individuals, but the energy expenditure is high [8, 9], making it unfeasible for daily use [10–12]. The recent emergence of powered exoskeletons [13] for over ground walking could change this.

Three powered exoskeletons for over ground walking have been approved for sale in North America, the ReWalk [ReWalk Robotics, Inc., Marlborough, MA [14]], Indego [Parker Hannifin Co., Macedonia, OH [15]], and Ekso [Ekso Bionics, Richmond, CA [16]]. With training, individuals with severe SCI and preserved arm strength to handle walking aids can achieve walking speeds from 0.03–0.71 m/s in the various devices, and cover distances averaging 98 m in 6 min (CI: 80-117 m) [17]. The large variation in speeds and distances are multifactorial, and have been associated with level and severity of injury, age, and training duration [18].

While reports on training in over ground exoskeletons are promising, with suggestions of important physiological changes [14], few have reported training-induced changes in the nervous system, except for transient (i.e., within a session) reduction in spasticity [19, 20] and pain [20], and no change in cortical activity as measured by EEG, or H-reflex excitability [20]. We know that other forms of walking training in people with chronic SCI, including treadmill-based robotic training, can induce changes in motor and sensory function [21–25]. The number of training sessions used in studies of over ground exoskeletons also vary largely from 5 sessions [26] up to 60 or more sessions [27, 28]. It remains unclear the training dosage required to reach a plateau in performance, with only 3 studies on the Ekso reporting the number of steps achieved in each session [20, 29]. The dosage of training is highly relevant for clinicians, and may be different for the different devices. Finally, very few have reported the performance of these devices on different terrain and environments such as outdoors and in the home.

Here, we report findings from a training program with the ReWalk, focusing on the progression in training, and the neuroplasticity induced by the training, defined as any change in performance that suggests modification in the strength of neural pathways. We further report results from preliminary testing of the device for use in a home-like environment. Portions of this data have been published in an abstract [30] and thesis chapter [31]. A companion paper [32] reports results from interviews with the participants, to capture their perspective on training and using the device.

## Methods

### Participants

Potential participants were a convenience sample between May 29, 2014 to July 30, 2018, including self-referred individuals, and those made aware of our study by clinicians in the community or local support groups (Spinal Cord Injury - Alberta). Potential participants contacted us and were screened by phone, then in-person. *Inclusion criteria:* chronic (≥1 year after injury), non-progressive SCI, body weight < 82 kg (to ensure safety of our trainers), lower extremity length appropriate for the ReWalk, uses the wheelchair as the primary mode of mobility, able to use forearm crutches, able to train for 4 days/week, and written approval for participation from a primary care physician. *Exclusion criteria:* comorbidities that interfere with training or measurements such as severe head injury, bone fractures within the last 2 years, low bone density (femoral neck t-score < − 3), hip and knee contractures > 10° flexion, ankle plantarflexion contracture, active pressure sores, severe spasticity, able to walk at a speed ≥0.4 m/s. Uninjured (i.e., control) participants were also recruited for comparison of some physiological measures. Participants were asked not to add new and regular activities during the training period. If they were already enrolled in a regular activity, they were welcome to continue. They were also asked to report any changes in medications. The study was approved by the Health Research Ethics Board at the University of Alberta (Pro00036789). Written consent was obtained from all participants (Table 1).

**Table 1:** Characteristics of participants with spinal cord injury

Participant code-gender	Age range	Neurological level of injury	AIS	Cause of injury	Time since injury (yr)	Anti-spastic medication
P1	2	T3	B	MVA	4.2	Baclofen
P2	1	C7	C	MVA	5.7	Baclofen, Tizanidine, Oxybutynin
P3	1	C6	C	MVA	2.5	Baclofen, Oxybutynin
P4	3	T6	A!	Crush injury	24.2	Baclofen, Diazepam, Oxybutynin
P5	2	T4	A!	Sports	16.2	Oxybutynin
P6	2	T3	A	MVA	4.3	Oxybutynin
P7	1	T7	A	MVA	2.4	Baclofen, Oxybutinin
P8	2	T9	A	Fall	1.3	Oxybutynin
P9	1	T10	B!	MVA	2.0	Baclofen
P10	2	T4^▲^	C^▲^	MVA	4.4	Baclofen, Oxybutinin
P11	3	C6	D!	MVA	18.7	Baclofen
P12	1	T7	A!	MVA	1.6	Baclofen
Mean^a^ (SD)	37.5 (13.7)				7.6 (8.1)	

Age ranges are used rather than exact numbers to avoid potential identification of individuals. Age range: 1 = 18–30 yr., 2 = 31–50 yr., 3 = 50–65 yr. The mean and SD for age were calculated from the actual age of each participant. AIS was that obtained in discharge summaries from rehabilitation centers unless otherwise indicated. AIS - ASIA Impairment Scale, T – thoracic, C – cervical, MVA - motor vehicle accident, SD - standard deviation. Symbols: ^a^Mean and SD do not include P6 who dropped out after 2 sessions of training. !AIS results not available from records or never performed, so AIS was estimated based on muscle strength scores and sensory perception. ^▲^ Evaluation occurred prior to occurrence of syrinx. Syrinx was operated on and resolved before participation in this study

### Experimental design

This was a prospective cohort study with a single, 12-week intervention. The majority of measurements were taken at least twice at baseline, then at 6 and 12 weeks of training, and between 2 and 3 months after the end of training (to suit the availability of participants). Some measures were taken weekly (see below).

### The exoskeleton

The ReWalk 2.0 was used initially. Velcro straps secured the torso, pelvis, and legs to the device, with footplates inside the shoes. After a skin abrasion on the foot was experienced by the first participant, updated footplates (called unilateral calf-holders) were used. The ReWalk was upgraded to Version 5.0 during the training of P7 and P8. The Velcro straps below the knee were replaced by knee brackets for the training of the last 3 participants. The mode of operation (i.e., standing, walking, stair climbing) was controlled with a wrist worn communicator, which signaled the computer, carried by the participant in a backpack. In the walking mode, an anterior tilt of a sensor on the pelvic band triggered motors at the hips and knees to generate steps. A variety of walking parameters were set with a computer program prior to walking, including the threshold for the tilt angle, swing phase time, delay between steps, and maximum flexion angles at the hip and knee. Forearm crutches were used for balance.

### Trainers

All trainers were trained and certified by ReWalk Robotics. One licensed physical therapist (DL) trained all participants, with 4 other trainers (2 of whom are physical therapists) substituting during DL’s occasional absence.

### Procedures

#### Standing in the standing frame

Participants (*n* = 4) who had not been standing weekly prior to training started by standing in a standing frame (EasyStand Evolv, Morton, MN) 5 days/week for 2 weeks before walking. The aim was to ensure tolerance of the upright position before walking. ReWalk training was initiated when participants tolerated two 30-min bouts of standing.

#### Donning and doffing the ReWalk

Participants transferred into the ReWalk, which was set on a piano bench with adjustable height to accommodate different leg lengths. They participated in the donning/doffing procedures, and were assisted as needed. Padding was used at the discretion of the trainer (Alpha Classic Gel Liner, WillowWood, Mt Sterling, OH, normally used to line prosthetic sockets). Skin integrity was checked before and after each training session, and more often if necessary.

#### Standing balance in the ReWalk

In the ReWalk, participants learned sit-to-stand, stand-to-sit transitions and balancing in standing. Balance tasks included lifting one crutch at a time, both crutches simultaneously, and preventing falls with the crutches in all directions. Walking began once participants could maintain balance while lifting one crutch for > 30 s.

#### Walking in the ReWalk

Participants started walking indoors on a smooth floor. Each step was initiated by a slight forward lean of the torso and a rapid return to upright to allow for foot clearance. Walking speed was increased by modifying the parameters of the ReWalk mentioned above, and by the participant initiating consecutive steps more quickly. Participants aimed to increase the walking speed and distance, and the number of uninterrupted steps in a sequence. They practiced turning while walking by using the crutches to change the torso orientation and pivot on the leg in the stance phase. Once participants were comfortable walking, they learned to control the wrist-worn communicator. When deemed safe by the trainer, other terrains were included: carpet, ramps, outdoors, and some attempted stairs and curbs. The trainer maintained contact-guard of the pelvic band from behind and assisted as needed, with a spotter in front throughout training.

### Outcome measures

#### Measures of training

The total step count, walking distance, average walking speed, steps per bout of walking without stopping, and duration of the session were documented at every training session. The number of consecutive steps was counted manually for each sequence of walking, and the average number of steps/bout was used to quantify walking skill, because novice walkers often unintentionally stalled the device with inadequate toe clearance. Assistance required for donning and doffing the device, and the walking skills practiced (see list in Table 2) were documented weekly by the trainer. Assistance level required for each skill was categorized as: independent, supervision, contact guard, minimal assistance, moderate assistance, or not achieved. The first 3 categories were considered as no assistance required. Simulated home tasks (Table 2) were tested near the end of training for those who were safe to perform those tasks, and again graded by the assistance level required. The Activities of Daily Living Laboratory in the Occupational Therapy Department in our Faculty was used. The items tested were: 1) Reach high cupboard – reach above shoulder level and remove an item from a shelf, 2) Reach low cupboard – reach below waist level to remove an item from a shelf, 3) Open and close refrigerator door – open the door, remove an item, place item on counter, close the door, 4) Use sink – take an item, move item to sink, stand at sink, turn on tap, wash an item, place item on drain, 5) Use stove – stand at stove top, place an empty pot with a single handle on the stove, turn the stove on and off, remove the pot from the stove top. For all tasks, the participants moved to the appropriate area (i.e., walk by engaging ReWalk motors, or otherwise move themselves). They were instructed to use any method that allowed them to accomplish the task.

**Table 2:** Number of participants achieving skills in ReWalk without assistance

#	Donning/Doffing	#	Walking	#	Home environment
6/11	Transfer to/from device	9/11	Sit-stand transitions	9/9	Reach high cupboard
10/11	Attach chest straps	10/11	Turn L & R 180°	0/9	Reach low cupboard
9/11	Attach thigh straps	11/11	Stop when walking	6/9	Open/close fridge door
2/11	Attach leg straps	11/11	25 steps - no stops	9/9	Use stove
1/11	Insert & extract foot	9/11	6 min – no rest	9/9	Use sink
		6/11	10 m on carpet		
		3/9	Up and down ramps		
		8/9	Use wrist controls		
		8/10	Concrete, asphalt, dirt, grass		

The Column # refers to the number of participants who could perform the task without assistance/number who were tested on the task. Differences in the latter number is because some individuals were not deemed safe to try the tasks

#### Clinical outcomes

##### Walking

Walking speed over 10 m was recorded during continuous walking in the ReWalk (i.e., modified 10-Meter Walk Test [10MWT]), because starting and stopping the device added unnecessary variability. The 6-Minute Walk Test (6MWT) was performed in a 40 m hallway. The 10MWT and 6MWT have been validated for individuals with SCI [33–35]. The Physiological Cost Index (PCI) was estimated during the 6MWT and an identical test during wheelchair propulsion as follows:

$$ \mathrm{PCI}\ \left(\mathrm{heart}\ \mathrm{beats}/\mathrm{m}\right)=\frac{\left(\mathrm{Active}\ \mathrm{HR}-\mathrm{Resting}\ \mathrm{HR}\right)}{\mathrm{Average}\ \mathrm{locomotor}\ \mathrm{speed}} $$

Heart rate (HR: beats/min) was recorded every 30 s with a HR monitor with Bluetooth connection to a cell phone (POLAR H7, Polar Electro Canada, Lachine, QC). Resting HR was the average HR during the last 2 min of a 5-min sitting period immediately preceding the active period, and Active HR was the last 2 min of walking or wheeling for 6 min. Walking speed was averaged over the 6 min. For the ReWalk, the device was donned before the measures in sitting. PCI was also measured for walking without the ReWalk in participants who could do so. The caveat with using the PCI for people with SCI is that the level of injury (i.e., at or above T6) could affect the sympathetic drive, and so the comparisons should only be made within a participant [36]. Hence, the relative effort of walking was expressed as a ratio of PCI for walking over PCI for wheelchair propulsion in the same individual. Finally, the maximum walking distance without a rest, for up to 1 h, was measured indoors on a smooth floor at the end of training.

##### Manual muscle strength

The Upper and Lower Extremity Muscle Strength (UEMS and LEMS) were estimated with the scale from the International Standards for Neurological Classification of Spinal Cord Injury (ISNCSCI) [37] by a physical therapist (DL), before and after training. Upper extremity tests were only performed for those with cervical lesions.

##### Spasticity

The Spinal Cord Assessment Tool for Spasticity (SCATS) [38] was used to estimate clonus, flexor and extensor spasms in the lower limbs. The scores from both lower extremities were summed (total score: no spasticity = 0; maximum spasticity = 18). A physical therapist not involved in the training (PJM) performed the measures weekly for the majority of the participants. The first two participants were scored by the training therapist (DL) monthly; their data showed us that more frequent measures were necessary, so their data were not used.

##### Pain

Daily rating of pain immediately before and after a training session was determined with a numerical rating scale between zero (no pain) and 10 (worst pain imaginable) [39]. Neuropathic pain over a week was estimated with the McGill Pain Questionnaire Pain Rating Index [40], completed prior to a training session once a week. Range of scores for the Pain Rating Index is 0 for no pain, to 78 for maximum pain.

#### Neurophysiological outcomes

##### Balance

Sitting balance was measured on a force platform (Model OR6-7-1000, AMTI, Watertown, MA), with feet unsupported and hands crossed over the chest. For the limits of stability, visual feedback of the instantaneous centre of pressure with 8 equally spaced targets in a circle [41] were displayed on a computer monitor, about 2 m in front of the participant. Participants leaned as far as possible towards each target in random order. For postural sway, participants sat on the force plate as above with eyes closed, and the trajectory of the centre of pressure was measured for a maximum of 30 s of sitting [42, 43] or until balance was lost. The tests were repeated several times on different days before training. The last 2 measures were averaged to represent the baseline.

##### Strength of sensory pathways

Skin sensation was measured by surface electrical stimulation (Digitimer DS7A, Hertfordshire, England) of the C3-S2 sensory key points [44] defined by ISNCSCI [37], using disposable electrodes. Single pulses at a stimulus frequency of 2–3 Hz, pulse width 0.5 ms, were applied from below threshold to a maximum of 10 mA, twice. Sensory threshold was the lowest current at which a tapping sensation was reported out of the 2 trials [44].

##### Strength of descending motor pathways

Single-pulse transcranial magnetic stimulation (TMS) (Magstim 200, Whitland, UK) was delivered through a double-cone coil placed at the vertex with current flowing in an anterior to posterior direction, to induce motor evoked potentials (MEPs) in the back extensor muscles bilaterally. Bipolar surface EMG electrodes (Kendall H59P, Mansfield, MA) recorded the responses at 8 vertebral levels spanning the injury (Cariga et al. 2002). Location of electrode placements were photographed on the first testing session, and used for verification in subsequent tests. Responses were recorded with the muscles at rest, and stimulus intensity at 60% (*n* = 1), 70% (n = 1) or 80% (*n* = 9) of maximum stimulator output (MSO) depending on the participant’s tolerance. Because the MEPs were variable at rest in the first 3 participants, in subsequent participants (*n* = 8), responses were recorded with background muscle contraction, elicited by a variety of maneuvers such as chair push-ups, arm raises, resisted back extension, and slight forward lean. The stimulus intensity for these trials was set to a level that produced a consistent response at rest and ranged from 50 to 70% MSO. The stimulus intensity was kept the same for all background contraction levels, and all testing sessions for each participant. Five evoked responses were averaged for each experimental condition.

### Data analyses and statistics

Data analyses for each of the measures are described below along with descriptive and/or inferential statistics. Smallest real change or clinically meaningful differences are included, where known. Where these differences are unknown, statistical tests, effect size (Cohen d, mean change/mean SD) and confidence intervals (mean ± 2 x standard error of the mean) are used.

#### Measures of progression in training

##### During training

The training data (see above) for each participant were lightly smoothed with a 3 point running average, then averaged across participants. The average across participants was fit to an exponential curve: y = a x (1 – e^(−t/c)^), where y is the measure of performance at a session, a is the estimated final performance reached, t is the training session number, and c is the time constant at which performance reached 63.2% of the value of a. The equation assumes that the initial performance is zero. This form of exponential curve is commonly used to characterize motor learning [45].

##### Pauses in training

To determine if breaks in training longer than 7 days resulted in degradation of walking, the training measures of walking skill (i.e., average number of steps in uninterrupted walking bouts) and walking endurance (i.e., total distance walked) from 3 sessions before and after the break were compared. In addition, outcome measures of walking and sitting balance at the end of training were compared with the follow-up measures taken 2–3 months after training to determine if there was retention of the skills gained from training. Paired t-tests were used for these comparisons.

#### Clinical outcomes

The weekly measures from SCATS and the Pain Rating Index from the McGill Pain Questionnaire were described using Group-Based Trajectory Modeling (GBTM), as the measures are repeated over many time points (i.e., time series), and GBTM is ideal for describing such measures to explore possible clusters of individuals who follow a similar trajectory of change over time [46]; it is not an inferential statistic. The GBTM is an unsupervised, statistical modeling method to approximate the trajectory of changes in discrete data over time, assuming that the population distribution of trajectories arises from a finite, unknown number of groups of individuals who follow distinct longitudinal trajectories. The approach allows us to determine, in a naturally heterogeneous population, whether there are subgroups that follow different trajectories over time. It has been used successfully in tracking the time-course of participation in people after stroke [47]. The Akaike information criteria [48] was used to estimate the relative quality of GBTM models in clustering the presumed trajectories, i.e., the relative amount of information lost by a GBTM model compared to other models, for a given set of data. A STATA procedure *traj* was used as a plug-in of STATA® 14.0 for GBTM (http://www.andrew.cmu.edu/user/bjones/). When groupings were identified, we further explored whether there were differences between the identified groups in terms of their initial scores on the respective measures.

#### Neurophysiological outcomes

##### Balance

Data on sitting balance were analyzed using custom written codes in LabVIEW (National Instruments Corp., TX) and Excel. Sway speed was the average speed estimated for 21 s, which was the shortest duration all participants successfully completed the trial with eyes closed, with slower speeds indicating better performance [49, 50]. Limits of stability was estimated as the maximum excursions in the fore-aft and left-right directions, again a commonly used measure [49], then summed to provide a single score. The scores at baseline, mid-training and immediately after training were compared using a repeated-measures ANOVA. In all statistical tests, *p* < 0.05 was defined as significant. For the repeated-measures ANOVAs, if sphericity was violated, the Greenhouse-Geisser correction was used. Post-hoc contrasts focused on specific contrasts only (see Results). SPSS was used for all ANOVAs, and Excel for Student’s t-tests.

##### Strength of sensory pathways

We reasoned that the perceptual thresholds may change at or just below the level of the injury for those with clinically complete SCI, because walking over ground with an exoskeleton engages muscles in the torso [51], which may require attention to sensory input from the torso, thus inducing plasticity in sensory input around the level of the injury. A repeated-measures ANOVA was used to compare the thresholds before and after training for 3 spinal levels: the level of injury, one and two levels below the injury. A 2-way repeated-measures ANOVA was used, with factors: time points and spinal level.

##### Strength of descending motor pathways

The peak-to-peak amplitude of the 5 MEPs evoked by the largest stimulation intensity at rest were averaged together for each thoracic level and then averaged across all levels for the right and left side separately. MEPs from the two baseline experiments were averaged together and compared to the MEPs after training with a Paired t-test. For trials recorded with a background muscle contraction, MEPs that had a corresponding background EMG between 10 and 40 μV, as measured in the 50 ms window before the TMS pulse [25], were averaged together and analyzed similar to the resting MEPs. Only individuals with more than 30 sweeps per testing session that fit the above criteria for background EMG were included.

## Results

### Participants

**Fig. 1:**
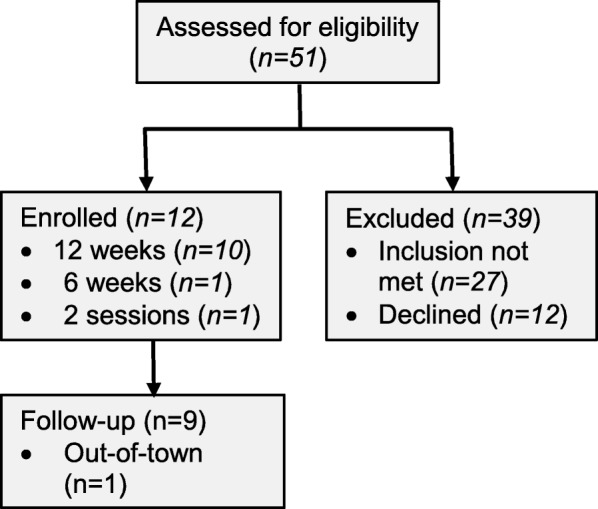
Participant flow chart. The number of potential participants screened, enrolled and followed are shown

Fifty-one potential participants were screened and 39 were excluded (Fig. 1). Twenty-seven of those excluded did not meet the inclusion/exclusion criteria, with the most common reasons being: over the weight limit (*n* = 6), contractures in the lower extremities (*n* = 5) and pressure sores (*n* = 5). Twelve others declined to participate, resulting in 12 eligible participants who were enrolled, 4 females (Table 1). Ten participants completed 12 weeks of training. One participant dropped out after two sessions of standing in the ReWalk. The only reason this participant gave was that it was not for them. As only baseline measures were available, the data from this participant were not included in the analyses. Another participant completed 6 weeks of training only, because an unrelated injury made it unwise to continue. Available data from this participant were included where possible. One participant who completed all training was lost to follow-up because of location of residence. During training, 8 out of 11 reported no change in activity level outside of the study. Three participants were engaged in other activities prior to training (2 in FES cycling and 1 in wheelchair rugby), but discontinued those activities during training either because of relocation or lack of time and/or energy for concurrent activity. One participant reduced their pain medication during the training because they found reducing it did not change their pain. This was not reported to us during the training, but became evident in the interviews [32]. While we know of no other changes, we cannot rule out possible changes that were unreported.

### Progression of training

**Fig. 2:**
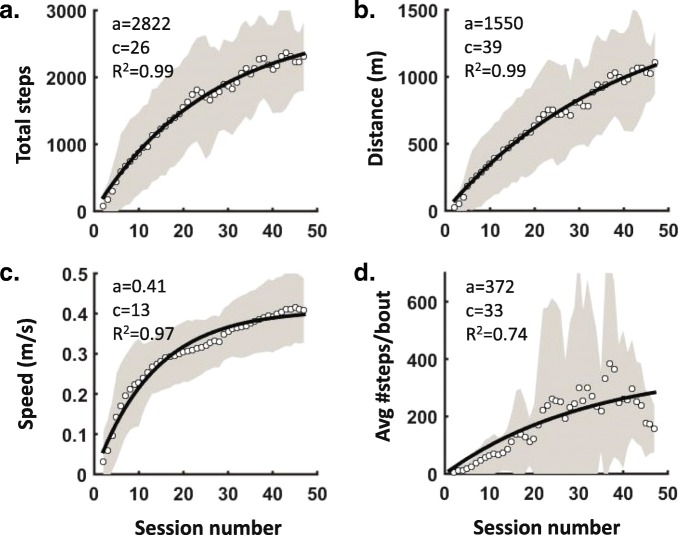
Progression in training. The measures recorded at each session include: **a**. total number of steps, **b**. total distance walked, **c**. average walking speed, and **d**. average number of steps in uninterrupted bouts of walking. This figure shows averages across all participants (*n* = 11) in the open circles, with one standard deviation shading. The best-fitting exponential curve (y = a x e ^(−t/c)^) is the solid black line, with the equation parameters and variance accounted for (R^2^) indicated. Equation parameters: t = session number, a = final performance, c = time constant, which is the session number at which 63.2% of the final performance is reached (see Data analyses for details)

The progress in walking training is shown in Fig. 2 for four performance measures obtained at each session: total number of steps (A), total walking distance (B), walking speed (C) and average number of steps in uninterrupted bouts of walking (D). The best-fitting exponential curves (solid lines in Fig. 2) are superimposed. The best-fitting constants representing final performance (a) and the time constant (c) are included in Fig. 2. Except for the two participants who dropped out, all other participants completed > 40 sessions of training (mean ± SD: 51.5 ± 6.0, range: 43–66) with an average frequency of 3.70 ± 0.2 sessions/week. By the end of training, all participants walked for about 1 h each session. The time constant, which is the number of sessions required to reach 63.2% of the final performance based on the best-fitting exponential equation, varied from 13 sessions for walking speed, to 39 sessions for walking distance. Individuals varied considerably between each other with respect to the rate of learning, as seen by the one standard deviation shading in Fig. 2. Once participants could walk hundreds of steps without the device stalling, more difficult skills were added, so the average number of uninterrupted steps declined after 40 sessions of training (Fig. 2d). Note that participants varied largely with respect to when they were ready to practice difficult skills. The fit of the exponential curves to the data were good to excellent, with the variance accounted for between 0.74 and 0.99 (R^2^ in Fig. 2). The number of training sessions required to reach 80% of the final performance based on the equations were 21 for walking speed, 42 for total number of steps, 63 for walking distance, and 53 for walking skill (average of 45 sessions).

There were 14 required pauses of training that were over 7 days long among 9 participants. The pauses were for issues including skin abrasions, device breakage, trainer injury, and holidays. These pauses were analyzed to determine if resumption of training was associated with a reduction in performance. The pauses ranged in duration from 10 to 61 days (mean 22 days), and did not affect the walking distance (Paired t-test, *p* = 0.32, effect size = 0.27) nor the average number of uninterrupted steps (Paired t-test, *p* = 0.29, effect size = 0.29). See Additional file 1: Figure S3. Indeed, the follow-up measures at 2–3 months after training showed little sign of reduction in ability in sitting balance and walking in the ReWalk (Table 3).

**Table 3:** Comparison of measures immediately after training and follow-up

Measure	End training (mean ± 1SD)	Follow-up (mean ± 1SD)	N	*P*-value
10MWT (m/s)	0.43 ± 0.11	0.42 ± 0.10	8^a^	0.13
6MWT (m)	146.3 ± 35.3	143.1 ± 33.9	9	0.34
Limits of stability (cm)	21.4 ± 12.6	21.2 ± 15.3	7!	0.89
Sway speed (cm/s)	1.96 ± 1.45	1.64 ± 0.72	7!	0.64

All comparisons were made with Paired t-tests. SD: Standard deviation; N: Sample size; 10MWT: 10-Meter Walk Test; 6MWT: 6-Minute Walk Test. ^a^One participant did not perform the 10MWT at follow-up because of back pain. !One participant could not sit unsupported, another did not have a measure for the end of training because of an unrelated injury

### Clinical outcomes

#### Walking in the ReWalk

**Fig. 3:**
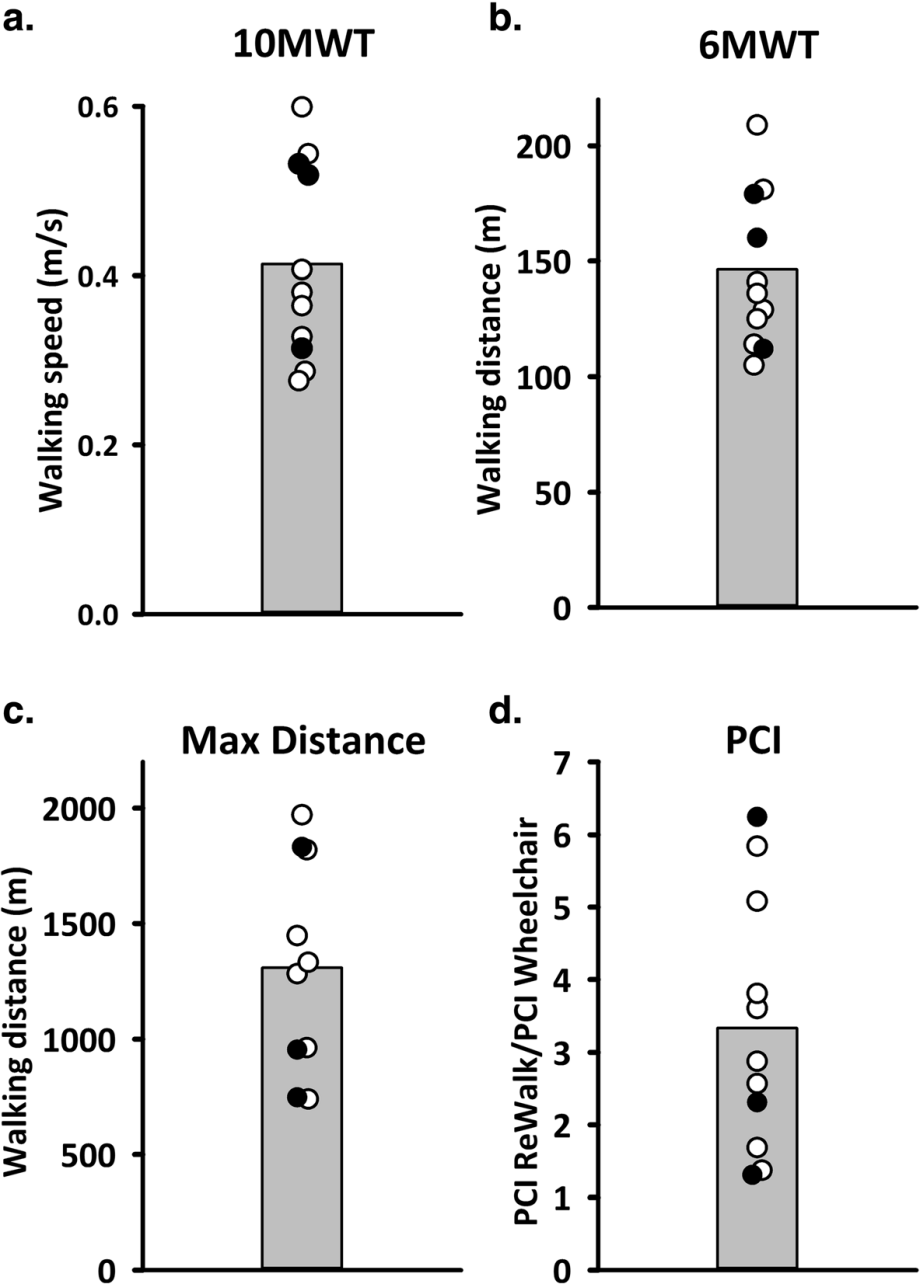
Walking measures at the end of training. The height of the bars represent the mean, with the individual participant scores in circles offset to show all data (n = 11). Filled circles represent those with motor incomplete injuries. **a**. Walking speed as measured during a modified 10-m walk test (10MWT). **b**. Distance covered during the 6-min walk test (6MWT). **c**. The maximum distance walked without a rest in 1 h. Ten data points shown here because P5 dropped out before a maximum distance was attempted. **d**. The effort of walking estimated with the Physiological Cost Index (PCI) while performing the 6MWT, expressed as a fraction of the PCI for the 6-min wheelchair test in the same person

Walking outcomes taken at the end of training are shown in Fig. 3, with each participant’s score shown in the circles, and the means represented by the height of the bars. No consistent differences were seen between those with motor complete (open circles) vs. incomplete (filled circles) injuries. The effort of walking in the ReWalk averaged across participants was a PCI of 1.60 ± 0.84 heart beats/m, which was 3.34 ± 1.75 times the effort of wheelchair propulsion in the same individual (Fig. 3d). The average effort for wheelchair propulsion was 0.49 ± 0.09 heart beats/m for the participants with SCI, similar to over ground walking for the uninjured participants (*n* = 7; age = 36.3 ± 13.3) at 0.52 ± 0.14 heart beats/m in a 6MWT. The distance walked was 675 ± 53 m for uninjured participants.

#### Walking without the ReWalk

Since P3 became a walker without the ReWalk after training, and P2 and P11 were able to walk short distances prior to training, their 10MWT, 6MWT and PCI without the ReWalk were also documented, using their preferred walking aid. All three walked further in the 6MWT (29 m, 89 m, 90 m further) and at a lower effort (PCI 38, 61, 80% less) with the ReWalk compared to without the ReWalk. P2 made some gains after training compared to before when not using the ReWalk, walking 14 m further in the 6MWT at a lower PCI (before training 3.5 heart beats/m, after training 2.3 heart beats/m). P11 did not change much without the ReWalk (4 m further at a PCI of 0.1 heart beats/m less). The smallest real difference in the 6MWT for people with SCI is 45.8 m [52]; similar information is not available for the PCI. So the gains made without the ReWalk did not reach the smallest real difference.

#### Skills

The number of participants attaining skills in the ReWalk without assistance are shown in Table 2. Without assistance was defined as not requiring physical assistance from the trainer (see Methods). All participants required some assistance with donning and doffing the device, especially with inserting and extracting the foot from the shoe and attaching the straps on the lower leg. Many walking tasks were possible for the majority of the participants without assistance, except for walking on carpet and ramps. Reaching high cupboards in the simulated kitchen was possible for most, while low cupboards were not, and managing a refrigerator door to extract items was challenging for some. Participants used a variety of means to accomplish tasks, such as leaning their crutches against the counters to free both hands, using one crutch to extend their reach (e.g., slide items along a counter or turn on a tap). Some could accomplish tasks at the counter using both hands, because they were sufficiently stable to stand without support, others leaned their body against the counter top to balance. To move around the tight space of a kitchen, many chose not to engage the ReWalk motors, but instead to shuffle their feet using both crutches to support body weight.

#### Pain

**Fig. 4:**
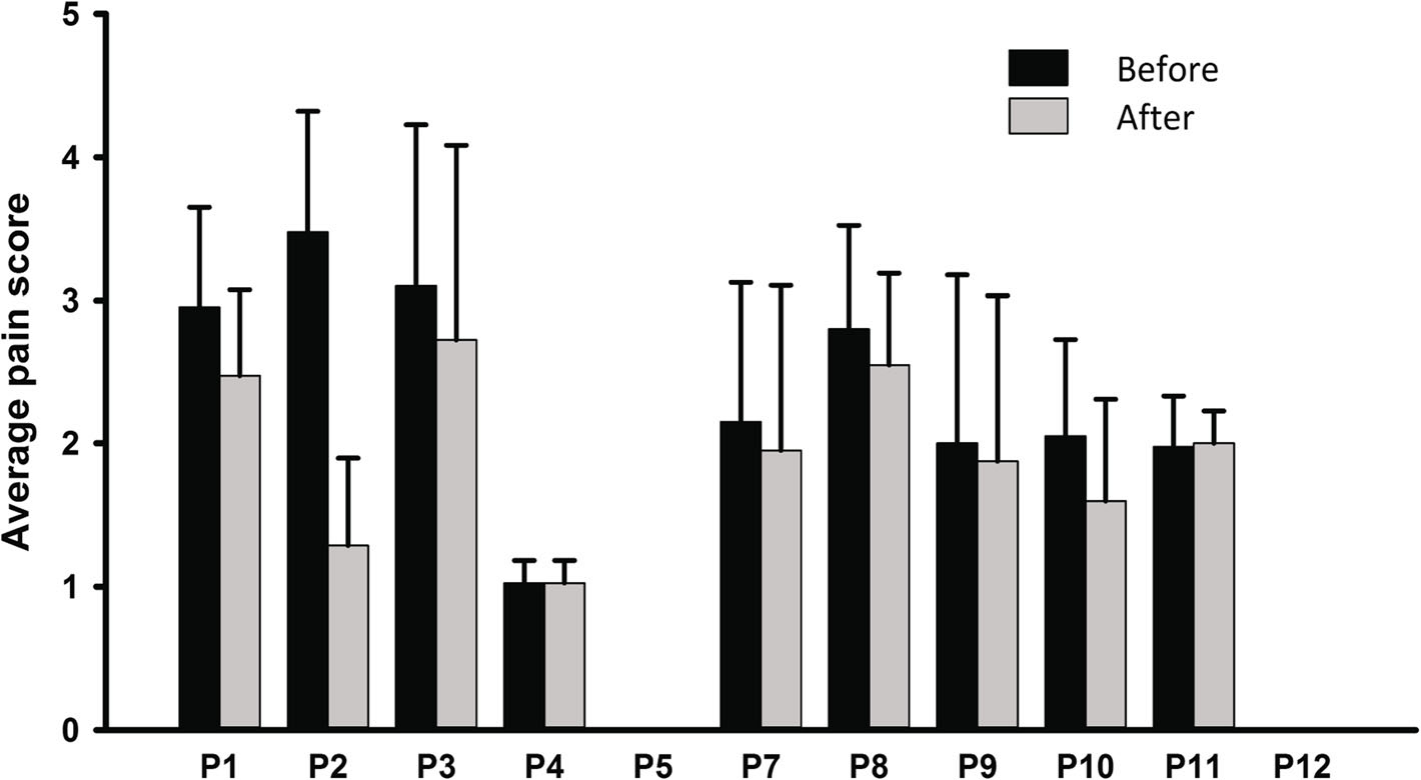
Average of pain scores before and after each training session for all participants. The numerical rating scale from 0 to 10 was used. P5 and P12 did not have neuropathic pain. The average with 1 SD is shown across the training sessions for each participant

**Fig. 5:**
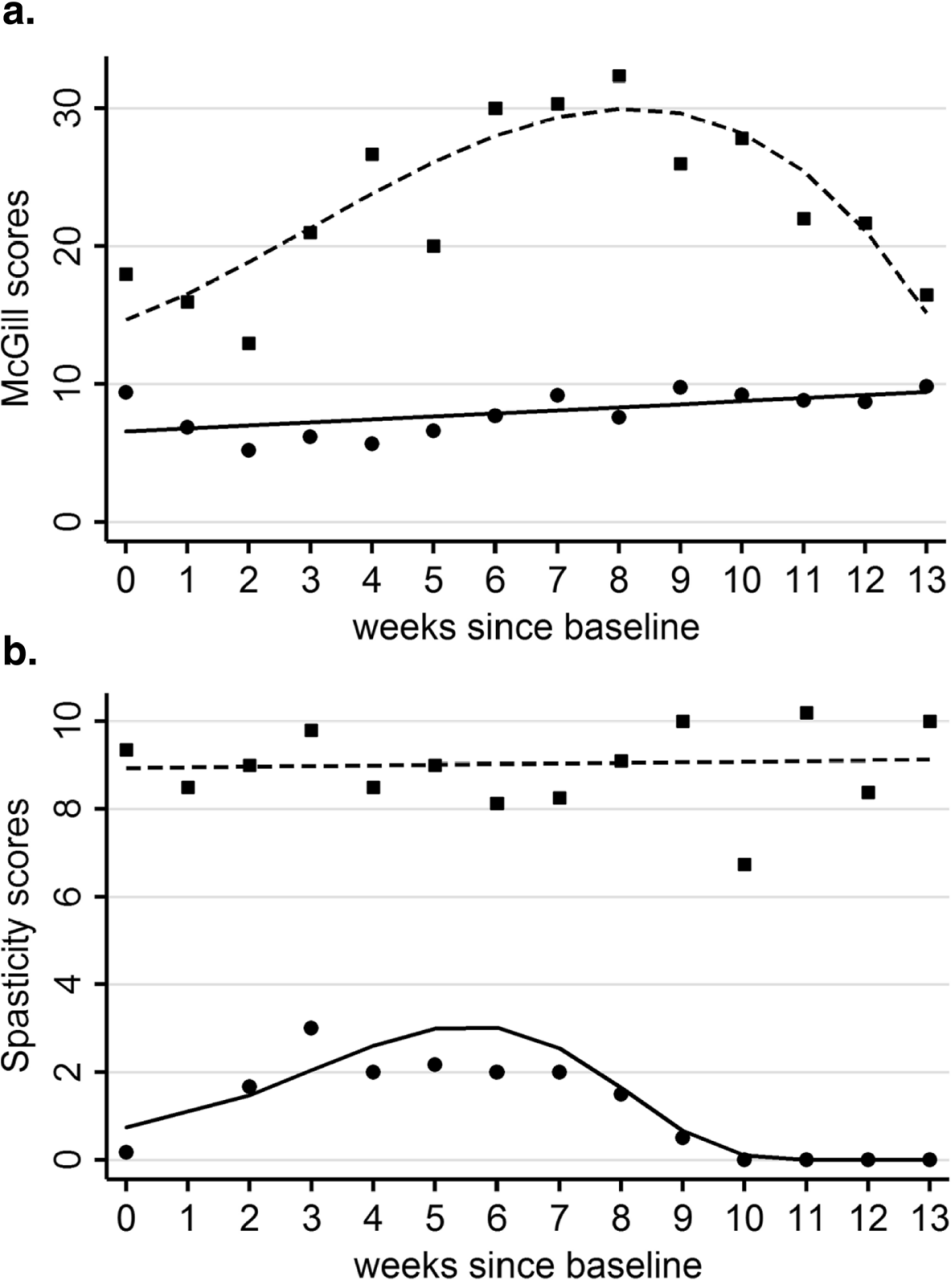
Results from Group-Based Trajectory Modelling (GBTM) of the weekly scores on neuropathic pain and spasticity. **a**. Neuropathic pain scores obtained from the McGill Pain Questionnaire. Dots represent the averages across participants in that group for each week starting from baseline (Week 0), and the lines represent the model. Participants with low initial pain scores showed minimal change over time (Group 1 – circles, solid line, *n* = 6). Participants with pain scores above 10 showed an increase then decrease to baseline (Group 2 – squares, dashed line, *n* = 3). Maximum score is 78. **b**. Spasticity from the Spinal Cord Assessment Tool for Spastic reflexes (SCATS), shown in the same format as **a**. Group 1 (*n* = 3, circles, solid line) consisted of participants with low initial spasticity (SCATS ≤0.5), who showed an increase then decrease in their spasticity. Group 2 (*n* = 5, squares, dashed line) consisted of participants with higher initial spasticity scores (SCATS ≥4.5), who did not show changes over time. Maximum score is 18

The Numerical Rating of Pain (scale ranges from 0 to 10) indicates participants tended to report lower average levels of pain after each training session compared to before they started the session. The real change in scores is smaller than a meaningful reduction in pain for persons with SCI, which is a change of 1.86 [53], with one exception (P2, Fig. 4). Sustained changes in pain were evaluated with the Pain Rating Index from the McGill Pain Questionnaire [40] (score ranges from 0 to 78) for 9 participants with scores over the 12 weeks of training. Meaningful reduction in the McGill Pain Questionnaire for people with SCI is unknown. The time course of change in these weekly scores was described using the GBTM (see Data Analyses), which suggested 2 groups with distinct trajectories over time and distinct initial scores (Fig. 5a). Group 1 was composed of 6 individuals with early McGill pain scores below 10; they showed minimal change over time. Group 2 included 3 individuals with higher pain scores (> 10); they showed a rise in the pain, and then a reduction back to baseline by Week 12. We emphasize that this analysis is exploratory and does not imply that these groupings might hold true for the population.

#### Spasticity

Eight participants had weekly measures on SCATS (score ranges from 0 to 18), which were also explored with the GBTM. The 3 sets of missing data were from the first 2 participants, who did not have weekly measures, and P5 who had incomplete measures because of leaving the study prematurely. The modelling suggested two patterns of change (Fig. 5b). Individuals in Group 1 (*n* = 3) with initial spasticity scores of 0 to 0.5 showed a small increase in the spasticity at the beginning of training, followed by a reduction back to near zero. Individuals in Group 2 with higher initial spasticity scores (mean ± 1SD = 9.4 ± 5.1; range 4.5 to 16.8) showed no change in spasticity over time. Again, the smallest meaningful change is unknown for this measure.

#### Muscle strength

Two out of 3 participants with motor incomplete injuries showed improvements in the Manual Muscle Strength Test for both upper and lower extremities (total score from both sides were: P3 UEMS increased from 30 to 37, and LEMS from 23 to 25; P11 UEMS increased from 33 to 37, and LEMS from 27 to 30, where the maximum score for UEMS and LEMS is 50). One of the two became a walker without the ReWalk. All other participants showed no change in muscle strength.

### Neurophysiological outcomes

#### Balance

**Fig. 6:**
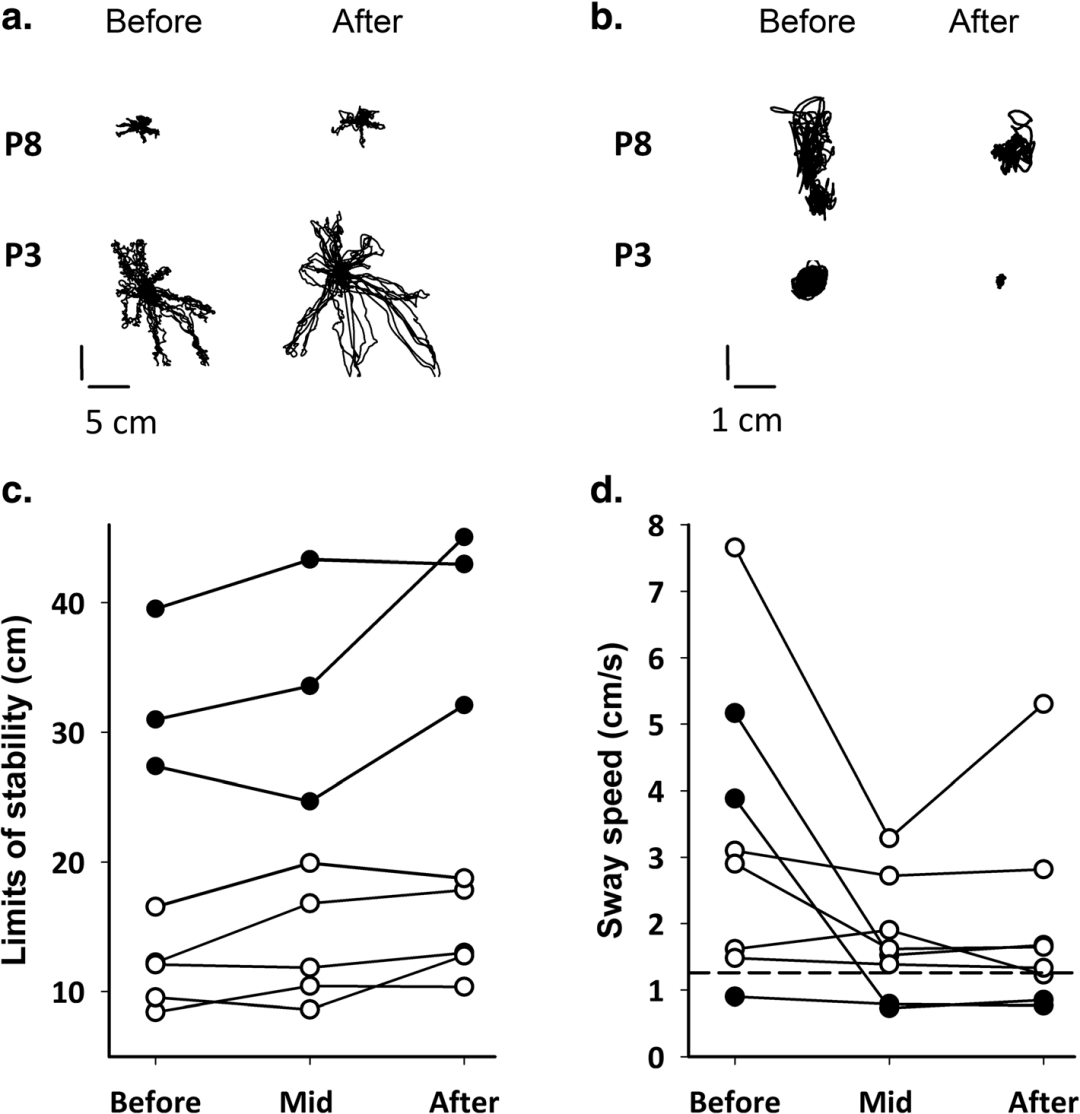
Sitting balance before and after training. **a**. Trajectories of the centre of pressure from the force platform during the test of limits of stability for 2 participants before and after training. The participants leaned in each of 8 directions with simultaneous visual feedback of the instantaneous centre of pressure and the targets at the perimeter of a circle. **b**. Trajectories of the centre of pressure from the same two participants during the test of quiet sitting with eyes closed. **c**. Group data showing the change in limits of stability before (Before), in the middle of (Mid), and at the end of training (After) for the 8 people with complete sets of data. The score in cm is the sum of the maximum fore-aft and medial-lateral excursion, with higher numbers indicating an improvement. Uninjured 51 ± 5 cm (*n* = 7; age = 35 ± 15 years, not shown). **d**. Group data showing the change in sway speed at the same time points as in **c**. Lower numbers indicate an improvement. Filled circles represent the individuals with motor incomplete injuries. The horizontal dashed line in **d**. indicates the mean sway speed for the uninjured participants (n = 7; age 35 ± 15 years)

Eight complete data sets are included for sitting balance, because one participant was unable to sit unsupported (P10), and two participants had incomplete data (P5 dropped out at mid-training, P12 had an unrelated fall out of the wheelchair causing rib pain on the day of testing at the after training time point), so the ANOVA results do not include these incomplete data sets. Figure 6a shows 2 individual examples of the centre of pressure trajectory during the testing of limits of stability, before and after training. They illustrate participants with small (P8) and large (P3) improvements. Limits of stability scores from individuals, consisting of the sum of the maximum fore-aft and left-right excursions, are shown in Fig. 6c, with solid circles representing people with motor incomplete injuries. For comparison, mean ± SD for uninjured controls was 51 ± 5 cm (*n* = 7; age = 35 ± 15 years). ReWalk training improved the limits of stability in sitting (repeated-measures ANOVA, with Greenhouse-Geisser correction F (1.53, 10.71), *p* = 0.03, observed power = 0.69). Students paired t-tests were used for the only 2 contrasts of interest (Before vs Mid-training, Before vs After training, hence significance with Bonferroni correction for 2 comparisons is *p* < 0.025). These comparisons showed the significant difference was between Baseline and After training (Paired t-test, *p* = 0.02, effect size = 1.09, CI = 12.8 to − 3.8 cm, mean change 4.5 ± 4.1 cm), not Baseline and Mid-training (Paired t-test, *p* = 0.13, effect size = 0.61, CI = 6.7 to − 3.6 cm, mean change 1.6 ± 2.6 cm). Sway speed in sitting with eyes closed also improved for many participants. Excursions of the centre of pressure during quiet sitting (eyes closed) are shown in Fig. 6b for the same two individuals as in Fig. 6a, with the sway speed calculated over 21 s from 8 participants in Fig. 6d. The change in average sway speed was statistically significant (repeated-measures ANOVA, with Greenhouse-Geisser correction, F (1.195, 8.367), *p* = 0.03 observed power = 0.62). Post-hoc contrasts indicated no differences between Baseline and After training (Student’s paired t-test, *p* = 0.03, effect size = 1.0, CI = -4.15 to 1.38 cm/s), as well as Baseline and Mid-training (Student’s paired t-test, *p* = 0.045, effect size 0.86, CI = 2.10 to − 5.29 cm/s). Uninjured controls showed a sway speed of mean ± SD = 1.26 ± 0.33 cm/s (*n* = 7), see dashed line in Fig. 6d.

#### Strength of sensory pathways

The electrical perceptual threshold was compared for people with motor complete injuries before and after training, at 3 levels – at the level of injury, 1 and 2 levels below the injury. Some reduction in thresholds (i.e., change in stimulation current at which a sensation was reported) were seen in 5 participants with motor complete injuries, and in one participant who had residual sensation at the S1 level (Additional file 1: Figure S1). Unfortunately, the clinically important change in current level is unknown. People with motor incomplete injuries (*n* = 3) are difficult to compare across individuals because of the diversity of their injuries, and also because their sensory thresholds were close to normal at many dermatomes.

#### Strength of motor pathways

All 10 participants who completed the training provided MEPs at rest. Since measures were obtained on each side and the injuries could be randomly asymmetric between people, we treated the left and right sides as independent. A Paired t-test showed no differences in resting MEPs evoked at the highest tolerable stimulation intensity before and after training (Paired t-test, *p* = 0.90, effect size = 0.03, *n* = 20, Mean ± SD 165 ± 177 μV before, and 162 ± 259 μV after training). Seven participants provided MEP data with sufficient number of trials across days with matched levels of background contraction (Additional file 1: Figure S2). Although the MEPs increased in two of the participants with incomplete injuries, the changes compared across the 7 participants was not significantly different (Paired t-test, *p* = 0.45, effect size = 0.21, *n* = 14, Mean ± SD 402 ± 193 μV before, 353 ± 118 μV after training). Background EMG values prior to the stimulus was also not different (*p* = 0.82, effect size = 0.06, Mean ± SD 22 ± 4 μV before, and 22 ± 3 μV after).

### Adverse events and technical issues

Two participants experienced a fall in training, during which the trainer controlled the fall, so no injuries were sustained by the participants. The thigh beam on the ReWalk 2.0 fractured just above the knee on 3 separate occasions, during which the trainer and spotter were able to prevent a fall. Near falls occurred occasionally, so the presence of a spotter and trainer was important.

Skin abrasions were experienced by six participants at skin locations in contact with the device, such as under the straps, the pelvic band, the knee bracket and footplate. Five required time away from the training to allow complete healing. One experienced a minor muscle strain from unexpectedly stalling the device, which resolved in two days.

The trainer behind the participant also experienced some minor injuries including biceps muscle strain precipitated by controlling a fall, first degree sprain of the knee during another controlled fall, and a bruised shin when the participant leaned too far forward during stance phase, causing the swing leg to hit the trainer at the time of toe-off.

## Discussion

All participants who completed training learned to walk in the ReWalk for about 1 km without a rest by the end of training, at speeds consistent with an indoor walker with spinal cord injury [54], and at an effort averaging about 3.3 times that of wheelchair propulsion. The rate of learning varied between participants, with an average of 45 sessions required across parameters to achieve 80% of the final performance. The number of participants in this study was small, and SCI is heterogeneous by nature, so the following discussion will focus on descriptive information with some use of inferential statistics.

### Training to achieve walking proficiency

Walking speed was most quickly learned by the participants, whereas walking distance and skill required more than 50 sessions to reach 80% of final performance (Fig. 2). There was considerable variability among participants with respect to the rate at which they learned to use the device, especially walking skill, as measured by the average number of consecutive steps without stalling the device (Fig. 2d). The differences in the rate of learning is presumably multifaceted.

In a large multi-center trial with the Ekso in Europe [55], 52 participants with either subacute or chronic SCI trained for 24 sessions. The average number of steps in each session showed a plateau at about 1000 steps near Session #18, with a walking time of about 25 min. At Session #18, our participants averaged 1359 ± 692 steps in one hour, and continued to improve in the following sessions (Fig. 2a). The European study suggested that by 24 sessions, a plateau in performance had been reached in the Ekso, whereas we found walking distance, steps and skill to continue to improve to beyond 40 sessions. The differences between the studies might be because the devices are different. For example, the Ekso provides greater support to the torso, so it may be easier to learn compared to the ReWalk. The differences could also be because of the larger number of training sessions in our study, allowing the participants to gain more endurance and skill. Suggestions consistent with the latter come from a longitudinal study using the HAL exoskeleton on the treadmill for 52 weeks of training, in which the walking distance and speed improved up to 12 weeks but not after [56]. The only caveat is that training in the HAL on the treadmill is very different from the ReWalk over ground. Given that our participants showed considerable variation in their progress, we recommend clinicians track each individual closely to identify when the performance reaches a plateau.

The participants indicated through semi-structured interviews, detailed in the companion paper [32] that learning to use the ReWalk was not as easy as they initially imagined. Many described the learning as a process of trial-and-error, consistent with implicit, motor learning.

### Powered exoskeletons for home and community

Participants walked at speeds of between 0.28 to 0.60 m/s in a 10MWT by the end of our study, which is comparable to other reports of similar powered exoskeletons for over ground walking [17, 18, 57]. The walking speeds achieved were between ‘supervised walker with outdoor wheelchair dependency’ (0.34 ± 0.1 m/s) and ‘walker indoor, wheelchair outdoor’ categories (0.57 ± 0.17 m/s) estimated by Van Hedel and colleagues for people with SCI [54]. Thus, the walking speeds are certainly functional, but insufficient (i.e., < 0.88 m/s) to be independent of a wheelchair.

The effort of walking in the ReWalk was 3.34 ± 1.75 times that of wheelchair propulsion in our participants (Fig. 3d). This effort is lower than that reported for reciprocating gait orthoses and hip-knee-ankle-foot orthoses for people with severe SCI, as reviewed in [17], and considerably lower than walking with Functional Electrical Stimulation (FES) and bracing (~ 11 beats/m) [58]. Oxygen consumption, a more direct measure of energy consumption, was reported to be 31% VO^2^_max_ in the ReWalk (11.2 ± 1.7 ml/kg/min at a walking speed of 0.22 ± 0.11 m/s) [59], and 51.5–63.2% VO^2^_max_ in the Indego (11.5 ± 1.4 ml/kg/min at a walking speed of 0.27 ± 0.05 m/s) [60], both lower than for FES walking at 70% VO^2^_max_ (16.19 ml/kg/min with walking speed not reported) [61]. Thus, walking in powered exoskeletons is not exceptionally energy demanding, which was corroborated by the impressions of the participants at the end of training, and certainly feasible for some individuals with SCI who are not otherwise ambulatory.

Walking skills such as turning while walking, walking on uneven surfaces, and on ramps were possible for the majority of participants with no assistance from the trainer (Table 2). Tasks in simulated home environments showed that many kitchen tasks were feasible, but maneuvering in tight spaces remained challenging, as stopping and turning require considerable space. The fact that a companion is needed to ensure safety is also limiting. Many positive perspectives on training were also recorded and detailed in the accompanying manuscript. Briefly, participants expressed other physiological and emotional benefits not documented by our quantitative measures.

### Neuroplasticity induced by the training

The training-associated changes in walking and balance suggest plasticity was induced in the nervous system, but it is difficult to separate neuroplasticity resulting from the use of uninjured pathways to support motor learning above the level of the injury, versus neural plasticity from changes in circuitry below the level of the injury. The methods used here cannot distinguish these different sources of plasticity. Presumably, those with incomplete injuries have greater potential for change compared to those with complete injuries.

The largest change was seen in P3, who has a motor-incomplete injury. Prior to training, this participant could stand only with the assistance of one person, and used a sliding board for transfers. After training, this participant could walk without the ReWalk at a slow speed (0.12 m/s) using a standard walker, and became independent in standing pivot transfers. These gains were in parallel with gains in muscle strength in the arms and legs (see Results). It is likely that the changes seen in P3 were a result of the ReWalk training, as this individual discontinued previous FES cycling during the period of ReWalk training. The improvements could have resulted from motor learning or recovery, or both. In two other participants with incomplete SCI (P2 & P11), training improved their walking speed without the exoskeleton, with gains of 0.08 m/s and 0.12 m/s, respectively in the 10-m walk test. These gains are very close to the smallest real difference for people with spinal cord injury of 0.05 m/s to 0.13 m/s [52, 62]. Conversion from a non-walker to a walker in one individual with a chronic, incomplete injury was also reported in the multi-center trial with the Ekso [55]. In addition, participants with motor-incomplete SCI who trained in the HAL showed average improvements of 0.22 m/s in the 10MWT without the device [63]. Thus, for people with motor-incomplete injuries, training in powered exoskeletons has the potential to improve muscle strength and function outside of the devices.

Most of our participants showed improvements in sitting balance (Fig. 6), but the power of the analyses were low (~ 0.6). Thus, these findings will have to be confirmed by future studies. The improvements observed are likely related to the use of the torso for balance during walking in exoskeletons over ground. Indeed, a recent comparative study of treadmill-bound versus over ground exoskeletons indicated more recruitment of trunk flexor and extensor muscles during walking in the over ground exoskeleton [51]. As many individuals showed some improvement in sitting balance, including those with clinically complete injuries, it is likely this improvement resulted from plasticity associated with motor learning. Improvements in sitting balance could lead to better function in other daily tasks, but this was beyond the scope of this study. Some participants indicated in the semi-structured interviews [32] that they noticed improvements in sitting balance, suggesting that for some, the improvements were meaningful. Surprisingly, the strength of the corticospinal tracts to back extensor muscles, as reflected by the size of the motor evoked potentials from single-pulse TMS showed no changes, although the statistical power was very low. It is possible that the MEPs recorded were dominated by the superficial muscles, such as the trapezius and latissimus dorsi, whose function is more related to movements of the neck, scapula and shoulder, rather than the control of the torso. Signal content from the deeper back extensor muscles may have been obscured. A better strategy for the future may be to record from some anterior muscles, such as the rectus abdominus, external and internal obliques. Alternatively, pathways besides the corticospinal tracts, such as reticulospinal pathways may be involved in the improvements.

Small improvements in skin sensation were observed in some individuals, but the changes were small and the smallest real difference in this measure is unknown. When changes were observed, they were typically in dermatomes just below the injury level and in skin regions with partial sparing of sensation. We speculate that these improvements may have been driven by the need to attend to all residual sensory input to successfully walk in the ReWalk, especially given the importance of maintaining balance during walking.

Neuropathic pain tended to be reduced after each training session for most participants (Fig. 5), but the magnitude of the reduction was small, below the smallest real difference [53] with one exception (P2 in Fig. 4). Small reductions in pain after each session of walking have also been reported for training in the Ekso [20, 64, 65], but long term reductions have not been reported [65]. Long term changes were mixed in our data set, as suggested by the GBTM (Fig. 5a) and would be useful to explore in the future.

Change in spasticity over time suggested those with initial SCATS scores of less than 2 showed a modest increase in spasticity followed by a return to baseline whereas those with initial scores greater than 5 showed no specific pattern of change over time (Fig. 5b). The small number of participants limit our ability to make conclusions, but the patterns would be interesting to track in a larger study or in meta-analyses if others also record these patterns over time.

### Device-related considerations

We used both the ReWalk 2.0 and 5.0, and thus observed some differences in their performances. The knee brackets included in the 5.0 version were much better for supporting the legs, as seen by a more extended knee position during standing and walking. While the incidence of skin abrasions was reduced while using the ReWalk 5.0, some were still unavoidable. The majority of participants also still needed some help donning and doffing the device at the end of training, with the task of inserting the feet in the shoes being most difficult (Table 2). A way to make this task easier or unnecessary would be helpful. For the purposes of using the device for rehabilitation, a more convenient adjustment to accommodate different pelvic sizes would help reduce operator time.

Many participants preferred not to use the wrist-worn controls, because it necessitated one crutch to be lifted off the ground to work the controls, which could be associated with trunk movement that could trigger a step before the crutch was returned to the ground and ready for walking. Locating the controls on the crutches may be a better alternative. The stair function was unsafe for our early participants, so we discontinued testing that function. We understand the stair function has been revised in ReWalk 6.0, which we did not have access to.

Fall prevention remains a problem [66], and to our knowledge, has not been addressed by any exoskeleton for over ground walking except the Rex, which is much heavier and slower [67]. The Ekso allows for an overhead tether, which is helpful in the early stage of training, but remains impractical if the device is to be used on other terrain or environments. Falls can occur even in experienced users, so it remains an important unresolved risk. We reduced this risk by always having 2 spotters, but this is not a good long term solution.

### Limitations

The number of participants in this study is small (*n* = 10 providing full data sets). The study is exploratory, and where statistics are included, they must be interpreted with caution. We did not correct the *p* values for the overall number of comparisons. We did not include a control group, based on the assumption that people with chronic injuries are not likely to improve spontaneously, but this remains a potential source of bias. Finally, there could have been other unknown biases, such as sampling bias, the participants changing their medication dosages without telling us, as we discovered from the semi-structured interviews in one case, reported in the companion paper [32].

## Conclusions

The ReWalk is a promising device to train walking in individuals with severe SCI with good upper extremity strength. The personnel required for training is substantial (i.e., average of 45 sessions with 2 trainers), balanced by benefits such as: ability to walk for long distances indoors and outdoors at a reasonable effort, improved sitting balance in some, and improved muscle strength in a few. While limitations remain, we feel that powered exoskeletons such as the ReWalk are making walking possible for many who previously were restricted to a wheelchair for mobility. We hope continued improvements to the devices will make them increasingly feasible for daily use and exercise in these individuals.

## Additional files

Additional file 1: Contains results from sensory testing, transcranial magnetic stimulation, changes in walking skill and distance after long breaks in training. (PDF 202 kb)

## Data Availability

The datasets supporting the conclusions of this article are included within the article and uploaded to Center for Large Data Research & Data Sharing in Rehabilitation.

## Abbreviations

10MWT: 10-Meter Walk Test
6MWT: 6-Minute Walk Test
FES: Functional Electrical Stimulation
HR: Heart rate
ISNCSCI: International Standards for Neurological Classification of SCI
LEMS: Lower Extremity Muscle Strength
MPQ: McGill Pain Questionnaire
PCI: Physiological Cost Index
SCI: Spinal cord injury
SD: Standard deviation
UEMS: Upper Extremity Muscle Strength
