# Supplementary material for: Retraining walking over ground in a powered exoskeleton after spinal cord injury: a prospective cohort study to examine functional gains and neuroplasticity
Source: J Neuroeng Rehabil. 2019 Nov 21;16:145. doi: 10.1186/s12984-019-0585-x (PMC6868817; doi:10.1186/s12984-019-0585-x)
Supplement: Supplementary file 1 — Contains results from sensory testing, transcranial magnetic stimulation, changes in walking skill and distance after long breaks in training. (PDF 202 kb) [file 12984_2019_585_MOESM1_ESM.pdf]

Supplementary Material

Changes in electrical sensory perceptual threshold

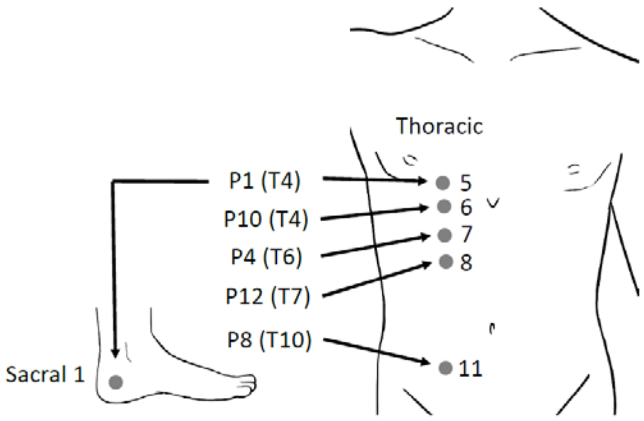

Figure S1. Location where sensory perceptual threshold was reduced after training compared to before training. Five participants showed small reductions in thresholds. The participant code and level of injury (in brackets) are indicated in text, with the actual level where change was recorded shown on the body sketches.

Change in motor evoked potentials elicited with single-pulse transcranial magnetic stimulation

Seven participants with matched levels of background EMG before and after training. The left and right sides for each participant is plotted separately.

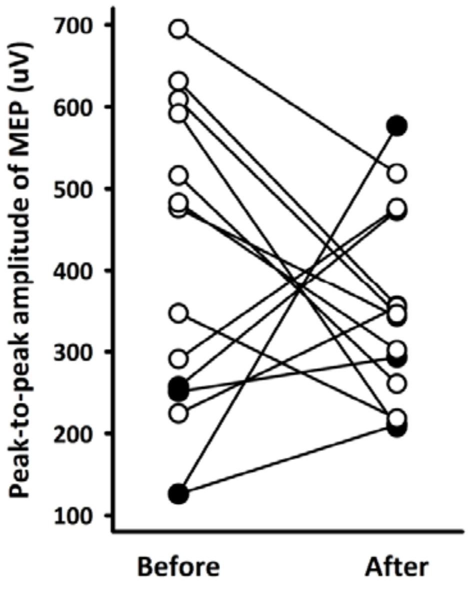

Figure S2. Average amplitude of peak-to-peak motor evoked potentials (MEPs) from back extensor muscles. Averages were calculated from trials with matched background EMG before and after training. Left and right sides from 7 participants are shown. Individuals with motor complete injuries are shown in filled circles. The differences before and after training were not significantly different (Paired t-test).

Effect of pauses in training greater than 7 days

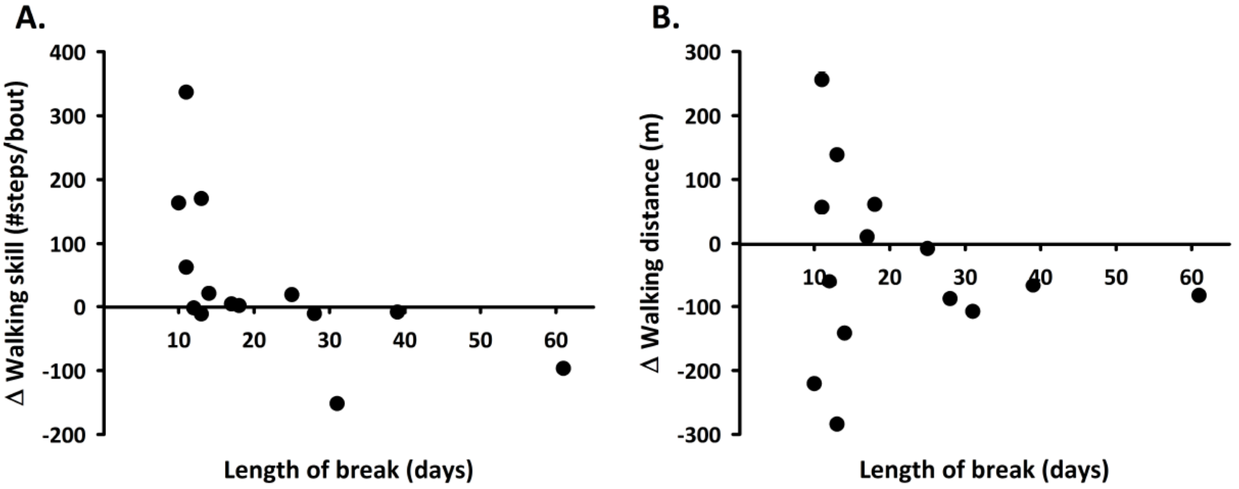

Figure S3. Change in walking skill (**A**) and distance (**B**) from measures taken before and after pauses in training of more than 7 days. Positive values represent improvements.
